# Supplementary figures and images for: Thiopurines inhibit coronavirus Spike protein processing and incorporation into progeny virions
Source: PLoS Pathog. 2022 Sep 19;18(9):e1010832. doi: 10.1371/journal.ppat.1010832 (PMC9522307; doi:10.1371/journal.ppat.1010832)

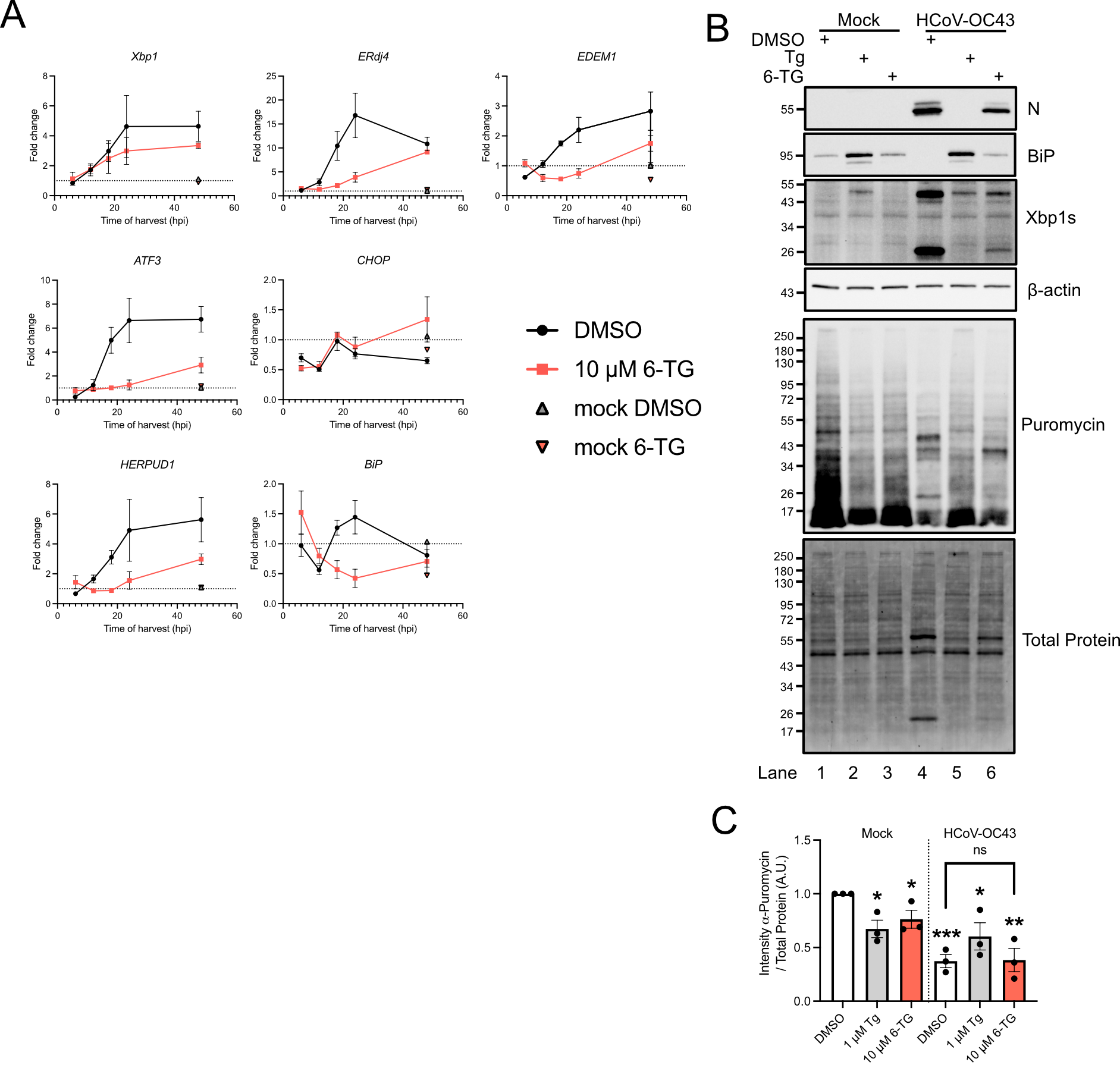

Supplement: S1 Fig — (A) 293T cells were infected with HCoV-OC43 at an MOI of 0.1 then treated with 10 μM 6-TG or DMSO vehicle control. Total RNA from the cells were harvested at the times indicated and stored at -80°C until RT-qPCR analysis of UPR target genes. (B) 293A cells were infected as in (A), then treated with 10 μM 6-TG, 1 μM thapsigargin (Tg), or DMSO. Cells were treated with 10 μg/mL of puromycin for 10 min prior to harvest 24 h after infection. Lysates were probed by western blot as indicated. (C) Total puromycin intensity from (B) was quantified relative to total protein load and are graphed normalized to the mock-infected, DMSO-treated cells (n = 3 ± SEM, statistical significance was determined by paired t-test compared to mock-infected, DMSO-treated cells; *, p<0.05; **, p<0.01; ***, p<0.001; ns, non-significant). (TIF) [file ppat.1010832.s001.tif]

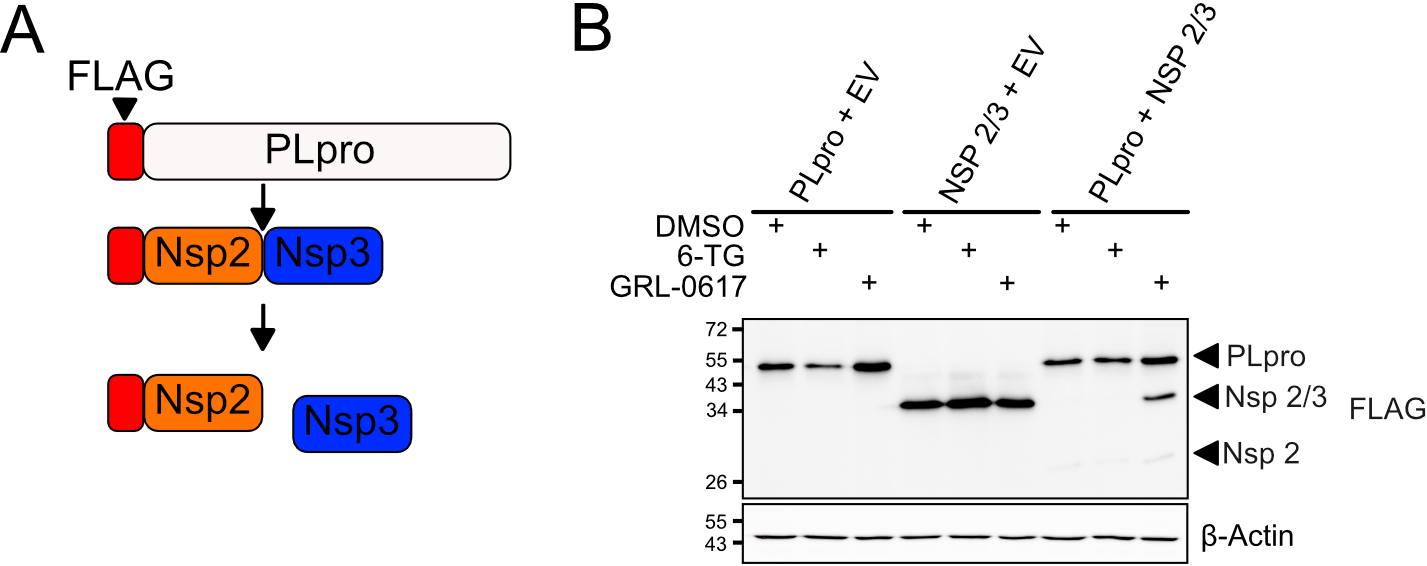

Supplement: S2 Fig — (A) Schematic of the cell-based PLpro protease assay. The 316 residue PLpro domain of SARS-CoV-2 Nsp3 was N-terminally FLAG (red) tagged and co-expressed in cells with an N-terminally FLAG tagged protease substrate comprised of the last 97 residues of Nsp2 (orange) and the first 100 residues of Nsp3 (blue). PLpro-mediated cleavage between Nsp2 and Nsp3 will liberate the Nsp3 portion from the FLAG-tagged Nsp2 polypeptide. (B) 293T cells were co-transfected with plasmids encoding SARS-CoV-2 FLAG-PLpro or FLAG-Nsp2/3 or empty vector as indicated then treated with DMSO vehicle control, 10 μM 6-TG, or 40 μM of the PLpro inhibitor GRL-0617. Lysates were harvested 24 h after transfection and probed by western blotting. (TIF) [file ppat.1010832.s002.tif]

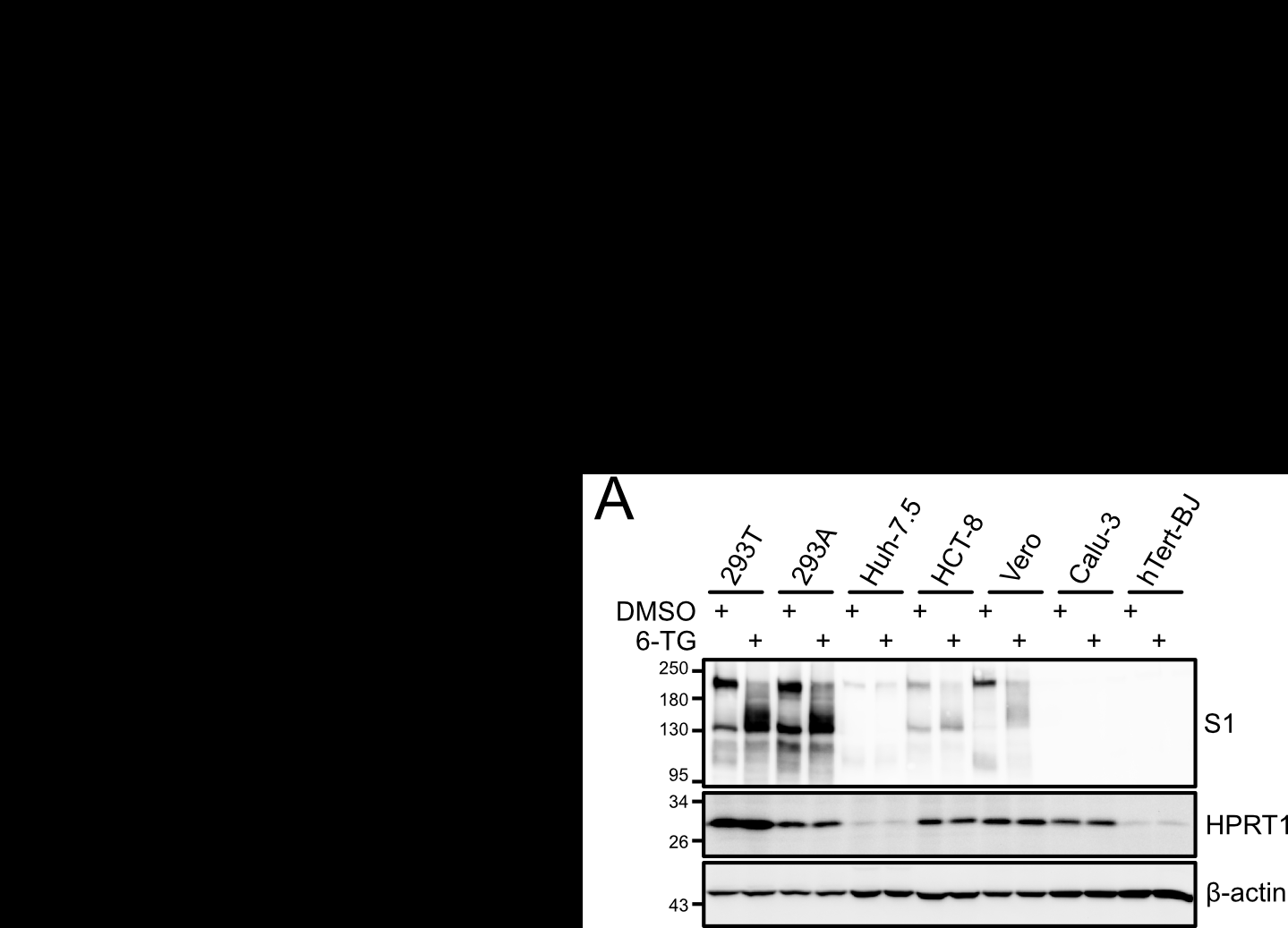

Supplement: S3 Fig — The indicated cell lines were transfected with plasmids encoding SARS-CoV-2 Spike followed by treatment with DMSO vehicle control or 10 uM 6-TG at 4 h post-transfection. Lysates were prepared at 24 h post-transfection and analyzed by western blotting as indicated. (TIF) [file ppat.1010832.s003.tif]
