# Supplementary material for: Thiopurines inhibit coronavirus Spike protein processing and incorporation into progeny virions
Source: PLoS Pathog. 2022 Sep 19;18(9):e1010832. doi: 10.1371/journal.ppat.1010832 (PMC9522307; doi:10.1371/journal.ppat.1010832)
Supplement: S1 Text — (A) 1H NMR spectrum of 2-amino-9-methyl-3,9-dihydro-6H-purine-6-thione. (B) 13C NMR spectrum of 2-amino-9-methyl-3,9-dihydro-6H-purine-6-thione. (C) High resolution mass spectrum of 2-amino-9-methyl-3,9-dihydro-6H-purine-6-thione. (DOCX) [file ppat.1010832.s004.docx]

**S1 Text:**

**Chemical synthesis of 2-amino-9-methyl-3,9-dihydro-6H-purine-6-thione (6-TG-Me)**

**General.** 6-Chloropurine was purchased from Toronto Research Chemicals (Toronto, ON, Canada). Thiourea and all other chemicals were purchased from Sigma-Aldrich Canada Ltd. (Oakville, ON, Canada). All NMR spectra were obtained using a Bruker AVANCE 500 MHz spectrometer. Chemical shifts (*δ* in ppm) for proton (^1^H) spectra **(Figure A)** are reported relative to the residual solvent signal for CDCl_3_ (*δ* 7.26), DMSO-*d*_6_ (*δ* 2.50), and HOD (*δ* 4.79)[^1^](#_ENREF_1). Chemical shifts (*δ* in ppm) for carbon (^13^C) spectra **(Figure B)** are reported relative to the residual solvent signal for CDCl_3_ (*δ* 77.16) and DMSO-*d*_6_ (*δ* 39.52).[^1^](#_ENREF_1) Abbreviations in NMR spectra are: bm, broad multiplet; bs, broad singlet; bt, broad triplet; d, doublet; dd, doublet of doublets; m, multiplet; q, quartet; s, singlet; and t, triplet. High resolution (HR) electrospray ionization (ESI) mass spectra (MS) **(Figure C)** were collected using a Bruker microTOF Focus orthogonal ESI-TOF mass spectrometer instrument operating in either negative or positive ion mode. Melting points are uncorrected.

**6-Chloro-9-methyl-9H-purine-2-amine (2).** Following a procedure adapted from published protocols,[^2^](#_ENREF_2)^,^[^3^](#_ENREF_3) 6-chloropurine-2-amine (2.0 g, 11.8 mmol) was dissolved in of dry DMF (100 mL). NaH (0.56g, 60 wt.%, 14 mmol) was then added and the suspension was stirred for 15 min before addition of iodomethane (13 mmol). The reaction mixture was stirred at room temperature for 2 h, then the solvent was evaporated *in vacuo*. The residue was dissolved in ethyl acetate and the solution was subsequently washed with water, dried over anhydrous MgSO_4_, and the solvent was evaporated *in vacuo*. The product was purified by silica gel chromatography (MeOH/CHCl_3_; 1:99) to afford 1.2 g of **2**. (56%). The ^1^H and ^13^C NMR spectra, as well as the mass spectrum, was in agreement with the literature.[^2^](#_ENREF_2)^,^[^3^](#_ENREF_3)

**2-amino-9-methyl-3,9-dihydro-6H-purine-6-thione (3).** Compound **2** (0.15 g, 0.82 mmol) was dissolved in ethanol (10 mL) followed by thiourea (0.25 gr, 3.27 mmol) and 2 drops of formic acid. The reaction mixture was the heated under reflux for 1 h. Upon cooling the reaction mixture to room temperature, a white precipitate formed, which was collected by filtration and washed twice with absolute ethanol (2 × 10 mL) to afford **3** as a white solid (120 mg, 81%); mp: >300 °C; ^1^H NMR (500 MHz, DMSO) *δ* 12.46 (s, NH, 1H), 8.56 (s, CH, 1H), 7.26 (s, NH_2_, 2H), 3.63 (s, CH_3_, 3H) **(Figure A)**; ^13^C NMR (126 MHz, DMSO) *δ* 173.28, 153.99, 147.09, 140.82, 123.44, 30.17 **(Figure B)**; HR-ESIMS: *m*/*z* calcd for C_6_H_7_N_5_Na_1_S [M+Na]^+^: 204.0314, found 204.0320. **(Figure C)**

**Figure A.** ^1^H NMR spectrum of 2-amino-9-methyl-3,9-dihydro-6H-purine-6-thione (**3**)

**Figure B.** ^13^C NMR spectrum of 2-amino-9-methyl-3,9-dihydro-6H-purine-6-thione (**3**)

**Figure C.** High resolution mass spectrum of 2-amino-9-methyl-3,9-dihydro-6H-purine-6-thione (**3**)


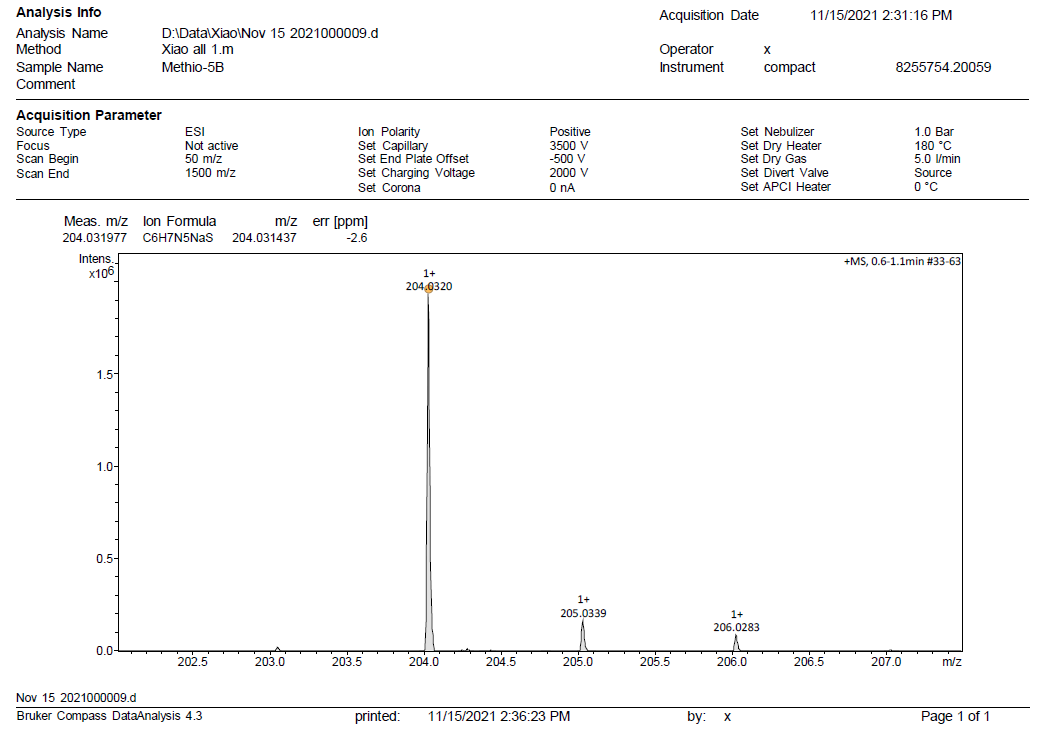


**REFERENCES**

(1) Gottlieb, H. E., Kotlyar, V., and Nudelman, A. (1997) NMR chemical shifts of common laboratory solvents as trace impurities. *J. Org. Chem.* *62*, 7512-7515.

(2) Norman, T. C., Koh, J. T., and Schultz, P. G. (1996) A structure-based bibrary approach to kinase inhibitors. *J. Am. Chem. Soc.* *118*, 7430-7431.

(3) Horejsi, K., Pohl, R., and Holy, A. (2006) Tricyclic purine analogs derived from 2-amino-6-chloropurine and 2,6-diaminopurine and their methylated quaternary salts. *Collect. Czech. Chem. Commun.* *71*, 77-90.
